# Synthesis of Highly Concentrated Suspensions of Silver Nanoparticles by Two Versions of the Chemical Reduction Method

**DOI:** 10.3390/mps2010003

**Published:** 2018-12-24

**Authors:** Miguel Gakiya-Teruya, Luis Palomino-Marcelo, Juan Carlos F. Rodriguez-Reyes

**Affiliations:** Department of Bioengineering and Chemical Engineering, Universidad de Ingenieria y Tecnologia—UTEC, Jr. Medrano Silva 165, Barranco, Lima 15063, Peru; mrgteruya@gmail.com (M.G.-T.); luispalomino026@gmail.com (L.P.-M.)

**Keywords:** Silver nanoparticles, UV-VIS spectrometry, dynamic light scattering, Frens method

## Abstract

In spite of the widespread use of the chemical reduction method to obtain silver nanoparticles, the nanoparticle yield is often low due to a required addition of small volumes of diluted metal ions to a solution containing a reducer. Higher yields can be obtained following an alternative method, in which the reducer is added to a greater volume of silver ions in the solution. In this study, protocols for both methods are detailed and compared, using characterization tools such as UV-vis spectrometry, dynamic light scattering (DLS), and zeta potential measurements. By using this alternative method, the amount of silver in the solution is three times greater, and nanoparticles with a narrower size distribution are formed (between 6 and 70 nm in size). In contrast, the regular method produces particles of 3 and 100 nm. Zeta potential measurements indicate that the nanoparticles synthesized with the alternative method will be more stable than those from the regular method.

## 1. Introduction

Metallic nanoparticles can be prepared using two approaches: top-down (when the starting point is a large portion of the material, which is down-sized) and bottom-up (when the nanoparticle precursors are ions or molecules which will nucleate and grow) [1]. The chemical reduction method, described in previous reports by other groups [2,3,4], is a bottom-up method that is relatively simple, which allows for the control of size and shape of nanoparticles. Nanoparticles produced with this method are stabilized by (i) maximizing the electrostatic repulsion between nanoparticles using capping agents (such as anions borohydride or citrate), and (ii) using small concentrations of metal precursors so that the likeliness of collisions of the growing nanoparticles are small. Thus, the stability of nanoparticles in suspension can be estimated by measuring the zeta potential (the electrostatic potential near the surface of a nanoparticle, which is derived from measuring the velocity of the particles in an applied electric field); greater values of zeta potential indicate greater repulsion and; therefore, an increased stability of nanoparticles [5]. The size and relative concentration of nanoparticles can be calculated using the dynamic light scattering (DLS) technique, where the scattered light produced by moving particles can be measured. The scattering pattern can then be related to the velocity of these particles and, in turn, to the size of the nanoparticles using the Stokes–Einstein equation [5].

The excessive growth of nanoparticle size is avoided by using low concentrations of metal precursors (in the mM range) resulting in low concentrations of nanoparticles. For demonstrative and educational purposes, it is accepted [2]; however, additional work with nanoparticles (for example, for functionalization or for studying interactions with biomolecules) [6,7] in higher concentrations may be desired. Surprisingly, there is a lack in the literature with respect to the comparison of both methods and the characterization and quantification of the resulting nanoparticles from the same laboratory. Below we report a protocol for obtaining a higher concentration of Ag nanoparticles in suspension, based on a modification of the Frens method [8]. We use UV-Vis and DLS to characterize the resulting solutions. A Nanodrop^®^ spectrometer is also used, as this type of spectrometer allows for the measurement of high concentrations of nanoparticles in suspension, as it can read higher absorbances than conventional spectrometers [9]. For comparison, we include also a protocol similar to the reported earlier by our group [10]. 

## 2. Experimental Design

### 2.1. Materials

- Silver nitrate (J.A. Elmer, 99.9%, Lima, Peru)
- Sodium citrate (Movilab, 99.9%, Lima, Peru)

### 2.2. Equipment

- UV-VIS spectrophotometer, ISR 2600 plus (Shimadzu, Kyoto, Japan)
- Nanodrop 1000 Spectrophotometer (Thermo Fisher Scientific INC., Waltham, MA, USA)
- Dynamic light scattering (DLS) Möbiuζ^®^ (Wyatt Technology, Santa Barbara, CA, USA)
- Vacuum Pump RV 3 (Edwards, West Sussex, UK)

## 3. Procedure

### 3.1. Synthesis of Silver Nanoparticles Using the Regular Method (Time for Completion: 1 h)

**Figure 1:**
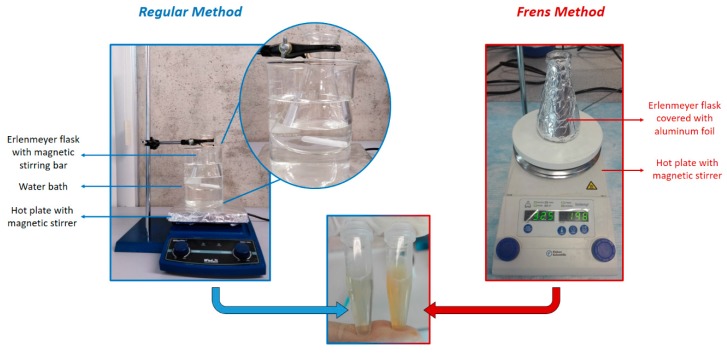
Setup for nanoparticle synthesis using the methods described in this protocol. The picture in the center compares the appearance of nanoparticle suspensions with these methods. Notice that the characteristic color of colloidal silver is more intense for the suspension obtained with the Frens method.

1. Place the solution of AgNO_3_ 2 mM on a dropper.
2. Place 50 mL of sodium citrate 7mM in an Erlenmeyer flask with a magnetic pill (see Figure 1).
3. Cover the flask with aluminum foil and heat it in a water bath until boiling point.
4. When the solution of sodium citrate has reached the boiling point add dropwise 8.8 mL of AgNO_3_.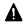 CRITICAL STEP: The solution of AgNO_3_ has to be added dropwise to obtain a good distribution of silver nanoparticles.
5. Stir the solution for 40 min.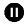 PAUSE STEP: After stirring, allow the suspension to cool down to ambient temperature.
6. Centrifuge 1 mL of the solution at 2040 RCF (relative centrifugal force, following the recommendation of the company Citodiagnostics [11]) for 30 min, discard the supernatant and resuspend in 1 mL distilled water.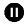 PAUSE STEP: The solution can be stored at 4 °C in the dark for up to 6 months.

### 3.2. Synthesis of Silver Nanoparticles Using the Frens Method (Time for Completion: 40 min)

1. Place 50 mL of AgNO_3_ 1mM in an Erlenmeyer flask with a magnetic pill (see Figure 1).
2. Cover the flask with aluminum foil and heat it until boiling point.
3. When the solution reaches the boiling point add 500 µL of sodium citrate 0.189 M (obtained from [12]).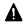 CRITICAL STEP: To obtain a good distribution and small size of silver nanoparticles, the concentration of the sodium citrate has to be as mentioned above.
4. Stir the solution for 20 min.
5. Centrifuge 1 mL of the solution at 2040 RCF (relative centrifugal force) for 30 min, discard the supernatant and resuspend in 1 mL distilled water.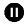 PAUSE STEP: The solution can be stored at 4 °C in the dark.

## 4. Results

### 4.1. Calculation of Nanoparticle Concentration

In this work we are reporting the use of two different amounts of silver nitrate (e.g., 50 mL AgNO_3_ 1 mM), which is mixed with 500 µL of sodium citrate 0.189 M. The concentration of silver in the final solution (in grams/L) can be estimated using the following formula:

M(Ag) = C(Ag) × V(Ag) × 108/V(total)

where M(Ag) is the weight concentration of silver in the final solution, C(Ag) and V(Ag) are the molar concentration (1 × 10^−3^ M) and the volume of the precursor solution (50 × 10^−3^ L), respectively, V(total) is the total volume of the suspension (the sum of volumes of silver nitrate and sodium citrate, 50.5 × 10^−3^ L) and 108 represents the atomic weight of silver (108 g/mol). Using the values from the Frens method, it results in a solution with a concentration of 0.107 g Ag/L (or 107 mg Ag/L). In the case of the regular method the concentration is 0.032 g Ag/L (or 30 mg Ag/L).

### 4.2. Characterization through Absorption Spectroscopy and Dynamic Light Scattering

**Figure 2:**
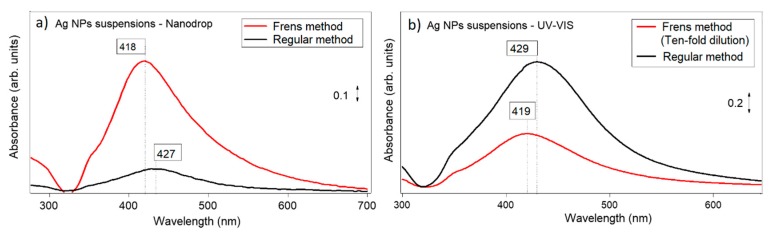
Absorbance spectra of Ag nanoparticles obtained with the regular method and with the Frens method, using a Nanodrop instrument (**a**) and a UV-Vis spectrometer (**b**). The suspension obtained via the Frens method exceeded the maximum measurement of the UV-VIS spectrometer (**b**); for this reason, the suspension was diluted by a factor of 10.

Silver nanoparticle suspensions using regular and Frens methods were characterized using UV-Vis spectrometry with a Shimadzu spectrometer and with a Nanodrop. The resulting spectra are shown in Figure 2 and the information is summarized in Table 1.

From the intensities of signals, it is clear that the Frens method produces suspensions with higher concentrations of nanoparticles. In addition, this method produces nanoparticles with a smaller diameter than those obtained with the regular method (lower absorption wavelengths are associated with smaller sizes of nanoparticles). Finally, from the analysis of the full width at half maximum, which is related to the dispersion of nanoparticle size, it is possible to see that the Frens method produces nanoparticles with a narrower size distribution.

**Figure 3:**
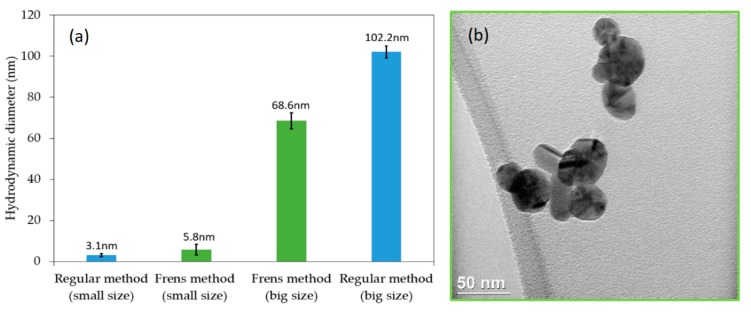
Determination of the nanoparticle size obtained by using the methods described in this protocol: (**a**) Using dynamic light scattering (DLS); (**b**) by transmission electron microscopy.

The broad spectra shown in Figure 2 suggest that nanoparticle suspensions are polydispersed, with contributions centered below 400 nm and above 450 nm. These positions are usually associated with nanoparticles that are smaller than 10 nm and above 50 nm. In order to confirm this suggestion, experiments using DLS were conducted and two groups of sizes were found, as shown in Figure 3. While with the regular method two size distributions are also obtained, around 3 and 102 nm, with the Frens method the two size distributions are centered around 5 and 68 nm. This observation agrees with the fact that the FWHM of spectra using the Frens method is narrower than the one obtained with the regular method. Figure 3b shows a TEM micrograph for Ag nanoparticles obtained using the Frens method, showing that “large” nanoparticles had spherical and cylinder shapes. Finally, the Zeta potential was also measured, finding a value of −1.3 ± 0.3 mV for the regular method and −27.7 ± 0.8 mV for the Frens method. Thus, the analysis of zeta potential suggests that suspensions prepared via the Frens method are more stable.

## 5. Summary and Conclusions

Two methods for the synthesis of silver nanoparticles were tested and compared in this study. Nanoparticle suspensions with higher concentration, improved stability, and smaller size distribution were obtained by a modified Frens method.

## Figures and Tables

**Table 1:** Comparison of intensity (arbitrary units, a.u.), position (nm), and full width at half maximum (FWHM, nm) for nanoparticles synthesized via the regular and Frens methods. Results from using UV-Vis spectrometry and Nanodrop are included.

	Peak Position (nm)		Intensity (a.u.)		FWHM (nm)	
	(Nanodrop)	(UV-Vis)	(Nanodrop)	(UV-Vis)	(Nanodrop)	(UV-Vis)
**Frens method**	418 ± 4	419 ± 3	0.9 ± 0.1	0.6 ± 0.5 *	116 ± 10	106 ± 5
**Regular metho**	427 ± 7	429 ± 5	0.2 ± 0.1	1.5 ± 0.6	123 ± 7	118 ± 6.1

* Sample was diluted (ten-fold).
